# Mortality in children and adolescents vertically infected by HIV receiving care at a referral hospital in Vitoria, Brazil

**DOI:** 10.1186/s12879-015-0893-0

**Published:** 2015-03-25

**Authors:** Sandra Fagundes Moreira-Silva, Eliana Zandonade, Angélica Espinosa Miranda

**Affiliations:** Infectious Diseases Department, Nossa Senhora da Glória State Hospital (SI-HEINSG), Vitória, Espírito Santo Brazil; Statistics Department, Federal University of Espírito Santo, Vitória, Espírito Santo Brazil; Postgraduate Program in Infectious Diseases, Federal University of Espírito Santo, Vitória, Espírito Santo Brazil; Núcleo de Doenças Infecciosas, Federal University of Espírito Santo, Av. Marechal Campos, 1468, Vitória, ES [29100-240] Brazil

**Keywords:** HIV, Children, Mortality, Late diagnosis, Causes of death

## Abstract

**Background:**

Daily throughout 2011, about 900 new HIV infections occurred in children and 630 children died as a result of AIDS-related complications worldwide. Late diagnosis, mortality trends, causes of and risk factors for death were evaluated in vertically HIV-infected children.

**Methods:**

A retrospective 11-year study was conducted with Brazilian vertically HIV-infected children and adolescents using patients’ charts. Medical records, death certificates and the Ministry of Health’s mortality database were verified for mortality and cause of death. Diagnoses were made according to the CDC Revised Classification System for HIV infection.

**Results:**

Of 177 patients included, 97 were female (54.8%). Median age at admission was 30 months (IQR: 5–72 months). Median follow-up was 5 years (IQR: 2–8 years). After 11 years, 132 (74,6%) patients continued in follow-up, 11 (6.2%) had been transferred and 8 (4.5%) were lost to follow-up. Twenty-six deaths occurred (14,7%), the majority (16/26; 61.5%) in children <3 years of age. Death cases decreased over time and the distribution of deaths was homogenous over the years of evaluation. In 17/26 (65.4%) of the children who died, diagnosis had been made as the result of their becoming ill. Beginning antiretroviral therapy before 6 months of age was associated with being alive (OR = 2.86; 95% CI: 1.12–7.25; p = 0.027). The principal causes of death were severe bacterial infections (57%) and opportunistic infections (33.3%).

**Conclusions:**

In most of the HIV-infected children, diagnosis was late, increasing the risk of progression to AIDS and death due to delayed treatment. The mortality trend was constant, decreasing in the final two years of the study. Bacterial infections remain as the major cause of death. Improvements in prenatal care and pediatric monitoring are mandatory.

## Background

Since 1996, when highly active antiretroviral therapy (HAART) began to be implemented and protease inhibitors were first introduced, a significant reduction has occurred in the morbidity and mortality associated with the human immunodeficiency virus (HIV) in adults and children [1-4]. Observational and clinical studies have confirmed a sharp drop in mortality rates in HIV-infected adults and children [5-9].

Nineteen years after the first study showed the efficacy of zidovudine in reducing the vertical transmission of HIV, failure to provide prenatal prevention continues in various resource-limited countries including Brazil [10-14]. Delays in the diagnosis of hundreds of HIV-infected children have increased their risk of progression to AIDS and death due to a lack of timely treatment [15]. A study comparing mortality in children with AIDS in the post-HAART era between developed and resource-limited countries showed the post-HAART mortality rates to be nine times higher in countries in which resources are scarce [16].

Daily throughout 2011, an average of 900 new HIV infections occurred in children and 630 children died as a result of AIDS-related complications worldwide, with the majority of these infections in the pediatric population being associated with vertical HIV transmission [17]. For more than ten years, efforts have been made to implement a “test and treat” strategy in pediatric HIV care [13]. The randomized clinical study, CHER (the **C**hildren with **H**IV **E**arly **R**etroviral Therapy Trial), on antiretroviral therapy strategies in South Africa showed that early diagnosis and prompt antiretroviral therapy in infants with vertically-transmitted HIV infection reduced early mortality by 76% and progression to AIDS by 75% compared to treatment in accordance with the consensuses [18].

According to the World Health Organization (WHO)’s recommendations [19], any HIV-infected child under two years of age should be treated with antiretroviral therapy as soon as possible, even when no symptoms are present and irrespective of CD4+ T-cell count. HIV-infected infants present clinical signs in the first year of life and without effective treatment one-third will die before completing one year of life and half will die before their second birthday [19].

Early diagnosis of the HIV infection will determine the prognosis both of children born to seropositive mothers whose condition was detected prior to or during pregnancy or delivery and of those with nonspecific constitutional symptoms, but who require recurrent medical care [4,13,14]. Children diagnosed as HIV-infected have a higher rate of infection and these infections are always more severe. Monitoring these children is important in order to be able to instruct the families on the severity of AIDS and its consequences, to provide better care to improve their quality of life and to increase their survival time [20].

A study conducted with HIV-infected children in Africa and in Brazil showed that the most important predictors of mortality in untreated children were CD4+ T-cell count, low weight for age and hemoglobin levels [21]. A cohort study of HIV-infected children and adolescents in Belo Horizonte, Brazil concluded that the most important predictive factors of progression to AIDS were advanced stages of the disease, CD4+ percentage < 15% and HIV viral load >5 _log10_ [22]. The objective of the present study was to evaluate the risk factors for progression to death, late diagnosis, causes of death and mortality trends in a cohort of vertically HIV-infected children and adolescents receiving care at a state referral hospital in Vitória, Espírito Santo, Brazil. The study population was monitored over a period of eleven years.

## Methods

A retrospective cohort study was conducted with vertically HIV-infected children and adolescents receiving care at the *Nossa Senhora da Glória* Pediatric State Hospital and followed up at the Infectious Disease and AIDS Pediatric Care Service in Vitória, Espírito Santo, Brazil. The study population consisted of all the patients with vertically transmitted AIDS receiving care at this unit between January 2001 and December 2011.

The mortality data on the patients being monitored and the basic cause of death were obtained from the medical records and directly from the death certificate. For patients who were lost to follow-up or who had been transferred to another service, data were obtained by consulting the mortality data system for the state of Espirito Santo during the study period, updated to December 2012. This database includes the death certificates collected by the State Health Department with the specific purpose of providing data of the greatest relevance for defining priorities in programs for the prevention and control of diseases [23].

The data were collected in 2011 and 2012 based on the patients’ medical records, using a specific standardized form on which the mother’s clinical information (prenatal care, whether the complete protocol for the prevention of the vertical transmission of HIV was applied, i.e. during pregnancy, delivery and in the newborn infant), aspects related to the family, demographic data on the child and its diagnostic status were recorded. In addition, data were collected on the quantification of baseline HIV plasma viral load, nadir CD4+ and CD8 T-cell count. Initiation of HAART before 6 months of age and for more than three months, as well as the presence of comorbidities classified according to the clinical classification categories defined by the Centers for Disease Control and Prevention (CDC, 1994) and the presence of congenital syphilis were also recorded [24].

With respect to the laboratory tests performed, between 2001 and 2008 viral load was quantified by reverse-transcription polymerase chain reaction assays (RT-PCR) using Cobas Amplicor® HIV-I Monitor™ test, version 1.5 (Roche Molecular Systems, Inc. Branchburg, NJ, USA), with a detection limit of 400 copies/mL. From 2009 onwards, the kit used was the b-DNA Versant® HIV-1 RNA 3.0 assay (Siemens, NY, USA), with a detection limit of 50 copies/mL. CD4+ T-cell count was measured by flow cytometry, using immunophenotyping (BD Biosciences, CA, USA). The Infectious Diseases Unit of the Federal University of Espírito Santo performed both tests using kits supplied by the Brazilian Ministry of Health.

The data were included in a spreadsheet using the Statistical Package for the Social Sciences statistical software program, version 18.0 for Windows (SPSS, Inc., Chicago, IL, USA). To evaluate mortality (the endpoint), the patients were stratified as dead or alive and the percentages of the qualitative variables were calculated for each group. The chi-square test or Fisher’s exact test was used as appropriate to measure the association between the qualitative variables and the endpoint. In the case of the quantitative variables, the means, medians, standard deviations and interquartile ranges were calculated and the Mann–Whitney non-parametric test was applied. For the study variables that were found to be statistically significant at a level of 10% in the chi-square test, logistic regression analysis was used to calculate the crude and adjusted odds ratios. Logistic regression analysis was performed in blocks, with the first block consisting of the sociodemographic and clinical variables and the second block the comorbidities. The forward stepwise likelihood ratio, in which the variables are included one by one to test for significance, was used to establish which of the variables were to be entered into the model. Only the adjusted odds ratios of the variables that remained in the final model will be presented. Significance level was established at 5%.

The internal review board of the *Nossa Senhora da Glória* State Pediatric Hospital (Reference Number 50/2009) approved this study. The data collected from the charts were used exclusively for the purposes of this study. The patients’ identity was maintained confidential through the use of codes.

### Consent

Written informed consent was obtained from the patient’s guardian/parent/next of kin for the publication of this report and any accompanying images.

## Results

A total of 177 children and adolescents with vertically transmitted AIDS were included in the study. Ninety-seven were female (54.8%) and the median age of the patients at admission to the study was 30 months (interquartile range [IQR]: 5–72 months). With respect to ethnicity, the majority was mulatto (n = 111; 62.7%) and 124 (70.0%) were from the metropolitan region of Vitoria.

**Figure 1:**
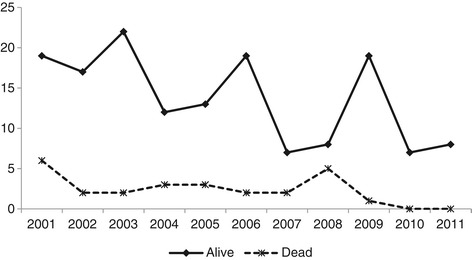
**Sample distribution, total number of children and adolescents and total number of deaths in absolute numbers according to the year of diagnosis of children and adolescents with vertically transmitted AIDS.** Infectious Diseases and AIDS Pediatric Care Clinic of the State Pediatric Hospital between 2001 and 2011 (n = 177), Vitoria, Brazil.

The total time of follow-up of the patients evaluated in this study was 11 years, with a median time of 5 years (IQR: 2–8 years). Of the 33 children who initiated antiretroviral therapy (ART) in 2001 and 2002, 25 are still alive and were monitored throughout the entire study duration. Two patients have never initiated HAART. At the end of the study, 132 patients (74.6%) remained in follow-up, 11 (6.2%) had been transferred to another service and 8 (4.5%) were lost to follow-up. Twenty-six patients (14,7%) had died (Figure 1).

The majority of these children were under three years of age (16/26; 61.5%) at the time of death, with deaths occurring throughout the entire decade evaluated. Figure 1 shows the distribution of the surviving and deceased patients according to the year of their diagnosis. Note a reduction in the number of cases over time and the even distribution of the deaths over the years of the study. The mean survival of the children included in the study was 10.20 years (95% CI: 9.62–10.79 years).

The majority of the patients who died were less than one year old when they were admitted to the clinic and most had advanced clinical and immunological disease (76.9%; 20/26). The majority had moderate/severe anemia (median hemoglobin level 9.7; IQR: 8.4 – 11.1) and had been on ART for more than three months (17/24; 71%). At admission to the specialist care service, the majority (20/26; 76.9%) of the patients who died were classified as clinical stage 4 according to the WHO staging system: the most severe stage and a predictor of mortality (p = 0.005). In 68.0% of the children who died, diagnosis was made as a result of them having become ill.

The association between the qualitative variables and the patients’ final status (alive/dead) is shown in Table 1. In relation to the endpoint (death), a statistically significant (p < 0.05) association was found with: age < 1 year at the time of admission to the service, the use of ART for more than three months, having advanced clinical and immunological disease, CD4+ percentage <15% and HIV plasma viral load ≥ 100,000 copies/ml, in addition to diagnosis having been made as a result of the patient becoming ill. Initiating ART before six months of age was associated with being alive (OR: 2.86; 95% CI: 1.12 – 7.25; p = 0.027).

**Table 1:** **Association between demographic and clinical variables, treatment, maternal HIV status, HIV plasma viral load and CD4+ T-cell count and mortality in children and adolescents with vertically transmitted AIDS**

				Current Status*				
**Variable**		**Total**		**Alive**		**Dead**		p-value**
		**N**	**%**	**N**	**%**	**N**	**%**	
**Sex**								
Male		80	45	71	47	9	35	0.240
Female		97	55	80	53	17	65	
**Age-group at admission**								
<1 year		60	34	43	28	17	65	**0.001**
≥1 year		117	66	108	72	9	35	
**Skin color**								
White		33	19	30	20	3	12	0.314
Other		144	81	121	80	23	88	
**Place of residence**								
Metropolitan region		123	69	107	71	16	62	0.340
Other		54	31	44	29	10	38	
**ART initiated before 6 months of age**								
Yes		34	20	25	17	9	38	**0.023**
No		134	80	119	83	15	63	
**More than three months of ART**								
Yes		161	96	144	100	17	71	**0.001**
No		7	4	0	0	7	29	
**Severe immune impairment**								
Yes		85	48	65	43	20	77	**0.001**
No		92	52	86	57	6	23	
**CD4+ percentage**								
**>25%**		**67**	**38**	**62**	**41**	**5**	**20**	**0.024**
**15–24%**		**61**	**35**	**53**	**35**	**8**	**32**	
**<15%**		**47**	**27**	**35**	**23**	**12**	**48**	
**Baseline HIV viral load**								
**≥ 100,000**		**102**	**58**	**80**	**53**	**22**	**88**	**0.001**
**<100,000**		**73**	**42**	**70**	**47**	**3**	**12**	
**Mother had prenatal care**								
Yes		95	55	81	55	14	56	0.933
No		77	45	66	45	11	44	
**Mother’s diagnosis**								
Prior to or during delivery		56	32	48	32	8	32	0.983
Following delivery		118	68	101	68	17	68	
**Mother received prophylactic treatment**								
Yes		39	22	32	21	7	28	0.469
No		135	78	117	79	18	72	
**During pregnancy**								
	Yes	25	14	20	13	5	20	0.386
	No	149	86	129	87	20	80	
**At delivery**								
	Yes	33	19	28	19	5	21	0.813
	No	140	81	121	81	19	79	
**Infant received prophylactic treatment**								
Yes		38	22	31	21	7	29	0.359
No		135	78	118	79	17	71	
**Whether the entire ACTG 076 protocol was implemented**								
Yes		12	7	10	7	2	8	0.807
No		163	93	140	93	23	92	
**Type of delivery**								
Vaginal		105	72	86	70	19	83	0.233
Cesarean		40	28	36	30	4	17	
**Breastfeeding**								
Yes		113	73	98	75	15	63	0.212
No		42	27	33	25	9	38	
**Category of exposure of mother**								
Injectable drug user		16	11	14	10	2	12	0.869
Sexual		134	88	119	88	15	88	
Transfusion		2	1	2	1	0	0	
**How diagnosis of case was made**								
Mother with AIDS became pregnant		7	4	4	3	3	12	**0.003**
Mother during prenatal care		30	17	30	20	0	0	
Mother at or after delivery, or father		61	35	56	38	5	20	
When the child became ill		75	43	58	39	17	68	
When a sibling was diagnosed		1	1	1	1	0	0	
**Current care-giver**								
Mother, father or other relative		149	85	126	84	23	92	0.298
Adopted or in an institution		26	15	24	16	2	8	
**BMI adequate for age**								
Yes		26	34	23	38	3	18	0.111
No		51	66	37	62	14	82	

*There are missing values in some variables. **p-value of the chi-square test. The numbers in boldface highlight the statistical significance.

Infectious Diseases and AIDS Pediatric Care Clinic of the State Pediatric Hospital between 2001 and 2011 (n = 177), Vitoria, Brazil.

For the quantitative variables nadir CD4+ and CD8 T-cell count, the means, medians, standard deviations and interquartile ranges were calculated and the Mann–Whitney non-parametric test was performed. No statistically significant differences were found between the patients who survived and those who died with respect to nadir T-cell count, as shown in Table 2. In the 26 patients who died, mean CD4+ count was 610.0 (IQR: 324 – 1222) and CD8 T-cell count was 1,152 (IQR: 778 – 2,394).

**Table 2:** **Association between death and nadir CD4+ and CD8 T-cell count in vertically HIV-infected children and adolescents**

**Variable**	**Statistics**	**Current status**		p-value*
		**Alive**	**Dead**	
	Median	877.0	610.0	0.158
Nadir CD4+ T-cell count	Maximum	4556.0	5189.0	
	Minimum	8.0	44.0	
	25th percentile	520.0	324.0	
	75th percentile	1352.0	1222.0	
	Median	1331	1152	0.538
Nadir CD8 T-cell count	Maximum	7318	6784	
	Minimum	58	408	
	25th percentile	845	778	
	75th percentile	2042	2394	

*Mann–Whitney non-parametric test.

Infectious Diseases and AIDS Pediatric Care Clinic of the Pediatric Hospital between 2001 and 2011 (n = 177), Vitoria, Brazil.

Table 3 shows the association between comorbidities and death. Statistically significant correlations with the endpoint were found for: *Pneumocystis jiroveci* pneumonia, thrombocytopenia, severe recurrent bacterial infections, any type of candidiasis, chronic anemia, HIV/hepatitis co-infection, wasting syndrome, extrapulmonary cryptococcosis, kidney disorders, tuberculosis, and lymphadenopathy > 0.5 cm at more than two sites.

**Table 3:** **Association between comorbidity and death in vertically HIV-infected children and adolescents presented in order of statistical significance according to the chi-square test**

	**Current Status**				
**Comorbidity**	**Alive**		**Dead**		**p-value**
	**N**	**%**	**N**	**%**	
*P. jiroveci* Pneumonia	21	14	13	54	0.000
Thrombocytopenia	15	10	9	36	0.002
Severe recurrent bacterial infections	63	43	19	76	0.002
Candidiasis – any type	45	30	15	60	0.004
Chronic anemia	94	63	23	92	0.004
HIV/Hepatitis co-infection	22	15	10	40	0.009
Wasting syndrome	29	19	10	40	0.023
Extrapulmonary cryptococcosis	1	1	2	8	0.054
Kidney disorders	1	1	2	8	0.056
Tuberculosis	17	11	7	27	0.056
Lymphadenopathy > 0.5 cm at more than two sites	106	71	13	52	0.057
First episode of pneumonia, bacteremia or sepsis	91	61	20	80	0.074
Prolonged diarrhea or gastroenteritis	57	38	14	56	0.095
HIV encephalopathy	19	13	6	24	0.138
Cerebral toxoplasmosis	1	1	1	4	0.148
Non-Hodgkin’s lymphoma	1	1	1	4	0.150
HIV-associated myocarditis	3	2	2	8	0.152
Splenomegaly	92	62	19	76	0.170
Persistent fever	46	31	11	44	0.196
Chronic parotitis	22	15	1	4	0.205
Lymphocytic interstitial pneumonia	31	21	3	12	0.304
Congenital syphilis	5	3	0	0	0.351
Any helminth or protozoan infection	14	9	1	4	0.374
Mucocutaneous herpes simplex	4	3	0	0	0.404
Dyslipidemia	11	7	1	4	0.532
Isosporiasis	2	1	0	0	0.556
Hepatomegaly	120	81	22	88	0.577
Dermatitis	63	42	12	48	0.593
Urinary infection	30	20	6	24	0.659
Progressive multifocal leukoencephalopathy	1	1	0	0	0.681
Disseminated mycobacteriosis	1	1	0	0	0.681
Acute otitis media or recurrent sinusitis	84	56	13	52	0.709
Cytomegalovirus infection	14	9	3	12	0.725
Pancreatitis associated with HIV infection	8	5	1	4	0.775
Congenital or acquired toxoplasmosis	10	7	2	8	0.902
Varicella or Herpes zoster	43	29	7	28	0.930
Gingivostomatitis or recurrent herpes	5	3	0	0	1
Cryptosporidiosis	1	1	0	0	1

Infectious Diseases and AIDS Pediatric Care Clinic of the State Pediatric Hospital between 2001 and 2011 (n = 177), Vitoria, Brazil.

According to the medical records, the causes of death (grouped according to diagnosis) were: severe bacterial infection (57.0%), opportunistic infections (33.3%), acute kidney failure (4.7%) and Burkitt’s lymphoma (4.7%). In five cases, the cause of death was unknown.

In the final regression model, the variables indicated as being most strongly associated with death were: being under one year old (adjusted OR: 10.20; 95% CI: 1.99 – 52.21; p = 0.005), *P. jiroveci* pneumonia (OR: 7.26; 95% CI: 1.62 – 32.54; p = 0.010), CD4+ T-cell count < 15% (OR: 4.25; 95% CI: 1.38 – 13.06; p = 0.012), chronic anemia (OR = 6.73; 95%CI: 1.53 – 29.64; p = 0.012), advanced clinical and immunological disease (OR = 4.41; 95% CI: 1.68 – 11.61; p = 0.003), severe recurrent bacterial infections (OR: 4.27; 95% CI: 1.61 – 11.32; p = 0.003) and tuberculosis (OR: 2.90; 95% CI: 1.07 – 7.92; p = 0.037) as well as extrapulmonary cryptococcosis (OR = 12.87; 95% CI: 1.12 – 147.70; p = 0.04) and kidney disorders (OR = 12.61; 95% CI: 1.10 – 144.72; p = 0.042) (Table 4).

**Table 4:** **Regression analysis for the risk of death in vertically HIV-infected children and adolescents**

**Variable**	**Crude OR**				Adjusted OR*			
	**p-value**	**OR**	**95% CI**		**p-value**	**OR**	**95% CI**	
**Age-group at admission to service**								
< 1 year	**0.001**	**4.74**	**1.96**	**11.46**	**0.005**	**10.20**	**1.99**	**52.21**
≥ 1 year		1.00				1.00		
**ART initiated before 6 months of age**								
Yes	**0.027**	**2.86**	**1.12**	**7.25**	0.860	0.86	0.16	4.63
No		1.00				1.00		
**Use of ART >3 months**								
Yes	a	a	a	a	a	a	a	a
No		1.00						
**Advanced clinical and immunological disease (B3C123)**								
Yes	**0.003**	**4.41**	**1.68**	**11.61**				
No		1.00						
**Percentage CD4+ T-cell count**								
>25%		1.00				1.00		
15–24%	0.296	1.87	0.58	6.07	0.244	2.62	0.52	13.22
<15%	**0.012**	**4.25**	**1.38**	**13.06**	0.107	5.06	0.71	36.29
**Baseline HIV viral load**								
≥ 100,000	0.004	6.42	1.84	22.36	0.115	3.31	0.75	14.71
<100,000		1.00				1.00		
**Origin of diagnosis**								
Mother had AIDS when she became pregnant		1.00				1.00		
Mother during prenatal care	a	a	a	a	a	a	a	a
Mother during or following delivery, or father	**0.017**	**0.12**	**0.02**	**0.69**	0.108	0.13	0.01	1.57
When the child became ill	0.247	0.39	0.08	1.92	0.532	0.49	0.05	4.64
At diagnosis of a sibling	a	a	a	a	a	a	a	a
**Comorbidities**								
*P. jiroveci* pneumonia	**0.001**	**7.20**	**2.85**	**18.19**	**0.010**	**7.26**	**1.62**	**32.54**
Thrombocytopenia	**0.001**	**5.02**	**1.89**	**13.33**				
Severe recurrent bacterial infections	**0.003**	**4.27**	**1.61**	**11.32**				
Candidiasis - any type	**0.005**	**3.47**	**1.45**	**8.30**				
Chronic anemia	**0.012**	**6.73**	**1.53**	**29.64**				
Hepatitis/ HIV	**0.004**	**3.85**	**1.53**	**9.65**				
Wasting syndrome	**0.027**	**2.76**	**1.12**	**6.77**				
Extrapulmonary cryptococcosis	**0.040**	**12.87**	**1.12**	**147.70**				
Kidney disorders	**0.042**	**12.61**	**1.10**	**144.72**	**0.020**	**50.92**	**1.84**	1411.87**
Tuberculosis	**0.037**	**2.90**	**1.07**	**7.92**				
Lymphadenopathy >0.5 cm at more than two sites	0.061	0.44	0.19	1.04				
Severe recurrent bacterial infection	0.082	2.51	0.89	7.05				
Prolonged diarrhea or gastroenteritis	0.099	2.05	0.87	4.84				

a: not calculated, no cases.

*Analysis performed with data from 153 cases (86.4% of the sample).

**This value is high due to problems in calculating the confidence interval, since there were few cases of this variable.

The numbers in boldface highlight the statistical significance.

Infectious Diseases and AIDS Pediatric Care Clinic of the State Pediatric Hospital between 2001 and 2011 (n = 177), Vitoria, Brazil.

## Discussion

The accumulated mortality rate was almost 15% for this cohort over the 11-year period of evaluation at a state referral hospital in Vitória, Espírito Santo, Brazil. This rate is higher than that found in a cohort of 320 children and adolescents in Belo Horizonte, Minas Gerais, Brazil (9.7%) and also higher than the rate of 12.1% reported in a study on the impact of HAART, opportunistic infections, hospitalizations and mortality in 371 children and adolescents in Belo Horizonte [8,9]. Nevertheless, this rate was lower than that of 26.9% found in the US Perinatal AIDS Collaborative Transmission Study (1986–2004) [4].

Analysis of the US perinatal HIV cohort showed a trend towards a gradual decline in mortality that corresponded to the advancements in antiretroviral therapeutics, with the mean annual mortality rate decreasing significantly between the three therapeutic eras. There was a reduction of over 60% in the number of deaths between the era in which no therapy or only monotherapy was available and the era of monotherapy or dual-combination therapy, and a reduction of 90% with the triple-combination therapy/antiretroviral therapy compared to the era of monotherapy/dual-combination antiretroviral therapy, with a notable increase in the proportion of deaths from causes unassociated with opportunistic infections [4]. On the contrary, in the present study, the principal grouped cause of death was severe bacterial infections, probably due to the differences between industrialized countries and resource-limited countries. As also reported in the study from Belo Horizonte, the two principal causes of death were pneumonia and sepsis [8]. In a cohort study conducted with 586 HIV-infected children in Thailand who initiated use of antiretroviral therapy, all the 42 deaths that occurred were attributed to infection [25].

In the present study, it is clear that the deaths were evenly distributed throughout the years evaluated (2001–2011). In 2011 in Brazil, the standardized coefficient of mortality in AIDS patients was 5.6 per 100,000 inhabitants; however, distribution varied over time [26].

When logistic regression analysis was used to adjust the OR for the risk of death in children and adolescents with vertically acquired AIDS in Vitória, a statistically significant association was found between death and the child being under one year of age at admission to the service (65.4%). At the time of death, the majority of the children were under three years of age. In the US cohort study, of the 364 HIV-infected children, 98 deaths occurred. Of these, 81% were under three years of age and 62% were under two years of age. The majority (84%) was born prior to 1994, three years before protease inhibitors became available as antiretroviral therapy (triple-combination therapy). Similar findings were reported in the French cohort of 348 HIV-infected children. Forty-seven of these children (13.5%) died before reaching their second birthday and 58 (17%) prior to their third. Most of the deaths occurred before 1996 [27]. In the present study, the cohort was followed throughout the decade of 2000, with the patients using dual-combination antiretroviral therapy and, later, triple-combination therapy in accordance with the Ministry of Health’s recommendations for the treatment of HIV-infected children.

The mean survival time of the children in this study was 10 years. Observational studies and international cohorts conducted to study survival in children with AIDS have reported a significant improvement, with results that are similar to those found in a Brazilian cohort showing an increase in the probability of survival of children with AIDS [4,11,25].

When the characteristics of the children and adolescents with AIDS acquired exclusively by vertical transmission, which represent 90% of the cases seen at this service, were evaluated, it was found that the majority had not been exposed to perinatal prophylaxis. This finding is in line with the statistics available on children with AIDS in Brazil. Of the 15,775 cases of AIDS in children under 13 years of age notified in the country up to 2011, 14,329 (90.8%) occurred by vertical transmission [26].

The main limitation of this study was the retrospective design. We could not recover some missing information. To minimize the limitations inherent to the design of the present study, a standardized form was used to collect the data. In addition, all the forms were reviewed and if any social data deemed important for the study were missing, information was requested from the family or caregivers.

At the time of admission to the service, most of the children who died in the present study were already classified as WHO stage 4, the most severe stage of the disease according to clinical and immunological criteria, this being a predictor of death (p = 0.005). In addition, in the majority of cases, diagnosis was reached as the result of the development of diseases that define AIDS, indicating that diagnosis of the HIV infection was late. The cohort study in Belo Horizonte, in which the prognostic value of the WHO’s clinical stage classification was evaluated in 335 HIV-infected children and adolescents, showed that patients at a risk of progression to AIDS or death were at clinical stage 3 and 4, with the risk of a poorer prognosis being greater in those at clinical stage 4. When controlled for CD4 count and viral load in a multivariate analysis, stage 4 remained an important predictor of death [22]. In a study conducted in Uganda in which 23,367 patients including 810 children and 575 adolescents were analyzed, a statistically significant association was found between mortality and advanced clinical stages of the disease according to the WHO classification system. The crude mortality rate was lower in children than in adolescents, a vulnerable and more complex group [28].

In agreement with the findings of other studies, the use of ART for more than three months, advanced clinical and immunological disease, percentage of CD4+ T-cell count < 15%, HIV plasma viral load ≥ 100,000 copies/ml and diagnosis of the case as a result of the patient’s illness were independent predictive factors of mortality. The study conducted with children in Belo Horizonte reported similar findings, with patients in whom CD4+ T-cell count was < 15% and viral load was 5 log being associated with a higher rate of adverse clinical progression and a greater risk of death [8]. In a double-blind, randomized, placebo-controlled study involving 566 symptomatic, HIV-infected infants and children in the United States aged between three months and eighteen years, Palumbo et al. evaluated the risk of progression of the disease based on baseline RNA levels and CD4+ T-cell count and showed that a combination of these two markers is capable of predicting progression of the disease and death. Furthermore, when treating HIV-infected children, suppression of viral replication enables the immune system to be maintained intact [29]. Monfenso et al. showed that viral loads of 100,000 copies and CD4+ T-cell counts <15% in children under 30 months old were independent predictors of an increased risk of clinical progression of the disease and death [30]. The longitudinal study, Cross Continents Collaboration for Kids (3Cs4kids), conducted with children over 12 months of age in Africa, showed that both CD4 percentage and absolute CD4 cell count were the greatest predictors of mortality. Furthermore, this study showed that mortality is high in underweight and anemic children of less than two years of age irrespective of their immunological status [21].

In agreement with those investigators, the results of the present study showed that moderate anemia was also significantly associated with mortality. On the other hand, no statistically significant association was found with respect to BMI for age. In the study conducted in Thailand, CD4+ percentage was independently associated with mortality, with an increased risk of 67% being reported for a decrease of 5% in nadir CD4+ T-cell count. Nadir CD4+ T-cell count, cachexia and hemoglobin levels were associated with a high risk of mortality even after the first year of antiretroviral therapy [25].

In the present study, the fact of having initiated ART prior to six months of life, having been diagnosed early and having initiated treatment promptly were found to be protective factors. These findings are in agreement with the results of the US study, in which HIV-infected children receiving ART in the first six months of life had a 94% likelihood of survival at 6 years of age [4]. The benefit of initiating antiretroviral therapy before reaching three months of life to avoid progression to AIDS and death in HIV-infected children has been shown in a randomized clinical study conducted in South Africa [18]. Furthermore, the European Collaborative Study concluded that initiating antiretroviral therapy prior to three months of life has a dramatic effect in reducing progression to AIDS and death in high-income countries [14].

In these eleven years of follow-up, loss to follow-up was only 4.5%, lower than that reported in other studies such as the 10.9% in the study conducted in Belo Horizonte and the 9.5% reported for the study carried out in France with perinatally-infected HIV-infected adolescents [8,27]. The median follow-up time until the final consultation was five years. Of the 33 children who initiated antiretroviral treatment in 2001 and 2002, 25 remained alive and in follow-up at the end of these eleven years. The median age at admission to the service was 30 months, characterizing late diagnosis of the HIV infection, a characteristic that has been reported in other Brazilian studies [15,31]. Unlike the findings of the present study in which diagnosis and treatment were late, in the cohort study conducted with perinatally HIV-infected French adolescents’ diagnosis of the HIV infection was made at an early stage and most of the children initiated treatment prior to nine months of life [27].

## Conclusions

In this particular Brazilian state, dozens of children have been diagnosed with HIV infection at a late stage, increasing their risk of progression to AIDS and death due to a lack of timely treatment. Mortality trends in HIV-infected children remained constant over the study period, with a decrease in the final two years. Bacterial infections remain the principal cause of death. Therefore, improving prenatal care and pediatric follow-up in an effort to diagnose vertically infected children as early as possible and treating malnutrition and anemia should be an integrated part of the healthcare provided to the child with AIDS, an action that may reduce mortality in these children.

## Abbreviations

AIDS: Acquired immunodeficiency syndrome
HIV: Human immunodeficiency virus
HAART: Highly active antiretroviral therapy
WHO: World Health Organization
OR: Odds ratio
IQR: Interquartile range
